# Transcriptomic and miRNA Signatures of ChAdOx1 nCoV-19 Vaccine Response Using Machine Learning

**DOI:** 10.3390/life15060981

**Published:** 2025-06-18

**Authors:** Jinting Lin, Qinglan Ma, Lei Chen, Wei Guo, Kaiyan Feng, Tao Huang, Yu-Dong Cai

**Affiliations:** 1 School of Life Sciences, Shanghai University, Shanghai 200444, China; 1290148376@shu.edu.cn (J.L.); mql1117@shu.edu.cn (Q.M.); 2 College of Information Engineering, Shanghai Maritime University, Shanghai 201306, China; lchen@shmtu.edu.cn; 3 Shenzhen Institute of Advanced Technology, Chinese Academy of Sciences, Shenzhen 518055, China; gw_1992@sjtu.edu.cn; 4 Department of Computer Science, Guangdong AIB Polytechnic College, Guangzhou 510507, China; kyfeng@gdaib.edu.cn; 5 Bio-Med Big Data Center, CAS Key Laboratory of Computational Biology, Shanghai Institute of Nutrition and Health, University of Chinese Academy of Sciences, Chinese Academy of Sciences, Shanghai 200031, China; 6 Department of Artificial Intelligence and Digital Health, CAS Engineering Laboratory for Nutrition, Shanghai Institute of Nutrition and Health, University of Chinese Academy of Sciences, Chinese Academy of Sciences, Shanghai 200031, China

**Keywords:** ChAdOx1 nCoV-19 vaccination, gene signature, miRNA signature, machine learning

## Abstract

Vaccination with ChAdOx1 nCoV-19 is an important countermeasure to fight the COVID-19 pandemic. This vaccine enhances human immunoprotection against SARS-CoV-2 by inducing an immune response against the SARS-CoV-2 S protein. However, the immune-related genes induced by vaccination remain to be identified. This study employs feature ranking algorithms, an incremental feature selection method, and classification algorithms to analyze transcriptomic data from an experimental group vaccinated with the ChAdOx1 nCoV-19 vaccine and a control group vaccinated with the MenACWY meningococcal vaccine. According to different time points, vaccination status, and SARS-CoV-2 infection status, the transcriptomic data was divided into five groups, including a pre-vaccination group, ChAdOx1-onset group, MenACWY-onset group, ChAdOx1-7D group, and MenACWY-7D group. Each group contained samples with 13,383 RNA features and 1662 small RNA features. The results identified key genes that could indicate the efficacy of the ChAdOx1 nCoV-19 vaccine, and a classifier was developed to classify samples into the above groups. Additionally, effective classification rules were established to distinguish between different vaccination statuses. It was found that subjects vaccinated with ChAdOx1 nCoV-19 vaccine and infected with SARS-CoV-2 were characterized by up-regulation of HIST1H3G expression and down-regulation of CASP10 expression. In addition, IGHG1, FOXM1, and CASP10 genes were strongly associated with ChAdOx1 nCoV-19 vaccine efficacy. Compared with previous omics-driven studies, the machine learning algorithms used in this study were able to analyze transcriptome data faster and more comprehensively to identify potential markers associated with vaccine effect and investigate ChAdOx1 nCoV-19 vaccine-induced gene expression changes. These observations contribute to an understanding of the immune protection and inflammatory responses induced by the ChAdOx1 nCoV-19 vaccine during symptomatic episodes and provide a rationale for improving vaccine efficacy.

## 1. Introduction

Coronavirus Disease 2019 (COVID-19), first identified in 2019, is caused by Severe Acute Respiratory Syndrome Coronavirus 2 (SARS-CoV-2) [1]. The rapid global spread of the disease has driven the accelerated development of vaccines, and vaccination with an effective vaccine can affect the body’s immune system, which, in turn, activates the associated immune response and induces the expression of immune-critical genes [2,3]. By reducing severe cases, hospitalizations, and transmission rates, COVID-19 vaccines have become essential public health tools in controlling the COVID-19 pandemic. ChAdOx1 nCoV-19 (AZD1222) is a recombinant vaccine developed by the University of Oxford. It consists of the replication-deficient simian adenovirus vector ChAdOx1, incorporating the full-length structural surface glycoprotein (spike protein) of SARS-CoV-2 along with a tissue plasminogen activator leader sequence [4].

Phase 1/2 clinical trials have demonstrated that a single dose of the ChAdOx1 nCoV-19 vaccine induces a robust Th1-skewed immune response, encompassing both humoral and cellular components. It can activate immune cells that secrete Th1-biased cytokines, produce Th1-biased IgG subclass (IgG1 and IgG3) responses, stimulate broad-spectrum T cell responses targeting S1 and S2 subunits of SARS-CoV-2 spike antigens, and induce SARS-CoV-2-specific IgM and IgA antibodies [5]. In addition, it can reduce the risk of disease exacerbations associated with Th2-dominant responses, showing significant protective efficacy. In a subsequent phase 2/3 trial, a second dose of the ChAdOx1 nCoV-19 (AZD1222) vaccine was found to not only enhance protective efficacy but also result in fewer adverse events after the second dose compared to the first [6]. A preclinical study showed that administering one or two doses of the ChAdOx1 nCoV-19 vaccine to rhesus macaques provided partial protection to their respiratory tracts, mitigating the severity of SARS-CoV-2 infection [7]. In addition, comparing the ChAdOx1 nCoV-19 vaccine with the BNT162b2 vaccine (another COVID-19 vaccine), it was found that the T cell responses following ChAdOx1 nCoV-19 vaccination were higher than those induced by BNT162b2 vaccination. In addition, a heterologous ChAdOx1-BNT vaccination schedule provides better protection against SARS-CoV-2 infection than two doses of ChAdOx1 nCoV-19 or BNT162b2 vaccine alone. The related immune genes induced by a heterologous ChAdOx1-BNT vaccination schedule have been identified [8,9,10]. These indicate that the safety, efficacy, and tolerability of the vaccine were demonstrated in multiple trials. The introduction of the ChAdOx1 nCoV-19 vaccine as a key measure to prevent the spread of the disease and reduce the burden on the healthcare system has significantly curbed the spread of SARS-CoV-2. In this context, the identification of immunomodulatory genes induced by the vaccine could help to gain insights into the molecular mechanism of the vaccine and provide a referable basis for improving the vaccine to enhance its efficacy.

In order to explore the key genes that could indicate the efficacy of the ChAdOx1 nCoV-19 vaccine and to further investigate the immunoprotective effect of this vaccine at the onset of symptoms and the immune memory before and after vaccination, the present study was conducted to obtain some samples related to the immunological efficacy of the ChAdOx1 nCoV-19 vaccine, based on the results of the study by Drury RE et al. [11]. These samples were obtained by RNA sequencing of blood collected from two groups of subjects who received different vaccines. One of the two groups of subjects was vaccinated with ChAdOx1 nCoV-19 vaccine as the experimental group, and one group was vaccinated with MenACWY meningococcal vaccine as the control group. After the grouping, five sets of samples were classified according to the different time points, vaccination status, and infection of SARS-CoV-2, which were the pre-vaccination group, ChAdOx1-onset group, MenACWY-onset group, ChAdOx1-7D group, and MenACWY-7D group. Both mRNA and small RNA profiles were obtained and analyzed in this study. Finally, the pre-vaccination group contained 29 samples, the ChAdOx1-onset group contained 33 samples, the MenACWY-onset group contained 47 samples, the ChAdOx1-7D group contained 30 samples, and the MenACWY-7D group had 41 samples. Each sample contained 13,383 RNA and 1662 small RNA features, and these data were used for subsequent analyses.

Traditional omics-driven studies usually focus on the overall integrated study of specific molecules in organisms, which combines with bioinformatics methods to systematically analyze the molecular features of biological systems or disease mechanisms, so as to discover biomarkers, functional genes, or regulatory networks. However, this research paradigm may have limitations, such as noise interference and a single analysis angle. These limitations restrict the mining of potential features [12]. Machine learning techniques that have emerged in recent years have effectively mitigated this limitation. In the face of the explosive growth of massive biological sample data, machine learning can identify potential markers or therapeutic targets from multiple perspectives, aiding in disease diagnosis [13]. This simplifies the identification steps, improves efficiency and accuracy, and facilitates subsequent experimental validation [14]. In this study, we designed a machine-learning-based framework, which integrated several machine learning techniques, to analyze the above-mentioned transcriptomic dataset on the ChAdOx1 nCoV-19 vaccine. We first used 10 feature ranking algorithms to analyze the data, yielding ten feature lists. Then, the incremental feature selection (IFS) [15] method combined with 12 classification algorithms was used to identify key features from each list, which can be used to understand the immune effects at different time points before and after vaccination with the COVID-19 vaccine. Machine learning algorithms can accelerate the prediction and identification of important genes or therapeutic targets associated with a target vaccine by automating the analysis of large datasets. Meanwhile, in order to avoid biased and unreliable results, the analysis process of this study involved 10 feature ranking algorithms and 12 classification algorithms. Through the analysis and validation from multiple perspectives, we finally screened the optimal classifier with a weighted F1 of 0.871 and extracted several essential RNA and small RNA features that were identified by multiple feature ranking algorithms. The relationship between the expression trends of these features and the ChAdOx1 nCoV-19 vaccine was discussed, confirming their special associations with the efficacy of ChAdOx1 nCoV-19 vaccine.

Finally, to solve the interpretability problem, we used a decision tree (DT) [16] with high interpretability to construct quantitative classification rules. On the one hand, these rules can be used to classify samples into five groups. On the other hand, they exhibited special patterns for different groups, giving new insights to investigate the efficacy of ChAdOx1 nCoV-19 vaccine. Compared to previous omics-driven studies, this study used emerging machine learning tools to analyze transcriptomic data, enabling faster and more comprehensive identification and study of ChAdOx1 nCoV-19 vaccine-induced gene expression changes, contributing to the further advancement of research on immune memory before and after COVID-19 infection.

## 2. Materials and Methods

### 2.1. Data

This study obtained a transcriptomic dataset from the research of Drury RE et al. [11], which was generated by collecting blood samples from participants and performing RNA sequencing. This dataset was also collected in Gene Expression Omnibus (GSE228839 and GSE228841). Based on the type of vaccine administered, the participants were divided into two groups: those vaccinated with the ChAdOx1 nCoV-19 vaccine and those vaccinated with the MenACWY meningococcal vaccine. The ChAdOx1 nCoV-19 vaccination group aimed to investigate the immune mechanisms underlying the body’s prevention of COVID-19 induced by the ChAdOx1 nCoV-19 vaccine, as well as to identify key genes that may serve as markers of ChAdOx1 nCoV-19 vaccine efficacy, while the MenACWY vaccine group served as the control group for the experiment. Subsequently, blood samples were collected from the participants at different sampling times, based on vaccination status and SARS-CoV-2 infection status, and divided into the following five groups: the pre-vaccination group (study participants who had not developed COVID-19 symptoms and had not received any vaccine), the ChAdOx1-onset group (participants who had received the ChAdOx1 nCoV-19 vaccine and exhibited COVID-19-related symptoms in the lungs but with uncertain nucleic acid test results), the MenACWY-onset group (participants who had received the MenACWY vaccine and exhibited COVID-19-related lung symptoms but with uncertain nucleic acid test results), the ChAdOx1-7D group (sampling data collected 7 days after the onset of symptoms in the ChAdOx1-onset group), and the MenACWY-7D group (sampling data collected 7 days after the onset of symptoms in the MenACWY-onset group). Of these five datasets, 29 samples were in the pre-vaccination group, 33 in the ChAdOx1 onset group, 47 in the MenACWY onset group, 30 in the chadOx1-7D group, and 41 in the menacWY-7D group. Details are provided in Table 1.

The collected blood samples were analyzed using next-generation (next-gen) sequencing, small RNA sequencing, and third-generation (3rd gen) sequencing, resulting in RNA-seq, small RNA-seq, and long-read RNA-seq data. Since the features of long-read RNA-seq and RNA-seq are similar, only the features from RNA-seq and small RNA-seq were used for further analysis. For RNA-seq data, Drury et al. aligned the sequencing data against the human genome GRCh38 using STAR v2.7.3a, and the gene counts were calculated using HTSeq v0.11.1. Genes with low counts across most samples were removed by only retaining genes with abundance greater than 3 counts per million in 9 or more samples. Ribosomal RNA (rRNA), sex chromosome genes, mitochondrial RNA, and haemoglobin genes were excluded from downstream analysis. For small RNA-Seq data, Drury et al. performed alignments using bowtie with up to 2 mismatches in the best strata mode and bowtie2 with 2 gaps permitted. Reads were first aligned to the non-coding transcriptome. The bowtie indexes for the non-coding RNAome were built by merging fasta files from the following databases: miRbase (miRNAs), SnOPY (snoRNAs), trRNA_db (tRNAs), and RefSeq (snRNAs, yRNAs, vault RNAs, lncRNAs). Non-aligned reads were then aligned to the mRNA transcriptome and antisense to the non-coding and mRNA transcriptome. All other reads were collapsed using a “gene_union” approach like those employed in seqCluster and mmQuant. Small RNA features were removed if they did not have an abundance of at least 5 counts per million in at least 20% of samples. The library sizes were normalized, and counts per million (CPM) were computed using the R package edgeR.

Ultimately, each group contains a different number of samples, with each sample containing 13,383 RNA-seq features and 1662 small RNA-seq features. A proper analysis of such features enables us to identify key characteristics that describe vaccine efficacy. This study also highlights the immune defense mechanisms and inflammatory responses induced by the ChAdOx1 nCoV-19 vaccine at symptom onset and analyze immune memory before infection, after infection, and after recovery (7 days+).

### 2.2. Feature Ranking Algorithms

A lot of features (RNA-seq and small RNA-seq features) were used to describe each sample. However, only a small proportion was related to describe vaccine efficacy. Several feature ranking algorithms were employed to investigate feature importance, thereby extracting key features. These algorithms included Least Absolute Shrinkage and Selection Operator (LASSO) [17], Monte Carlo Feature Selection (MCFS) [18], Minimum Redundancy Maximum Relevance (mRMR) [19], Categorical Boosting (CatBoost) [20], Extreme Gradient Boosting (XGBoost) [21], Adaptive Boosting (AdaBoost) [22], random forest (RF) [23], Extreme Randomized Tree (ExtraTrees) [24], Light Gradient Boosting Machine (LightGBM) [25], and ridge regression (Ridge) [26]. These algorithms are designed using different principles. In detail, LASSO and Ridge are linear regression algorithms and evaluate feature importance according to their coefficients. MCFS, CatBoost, XGBoost, AdaBoost, RF, ExtraTrees, and LightGBM are all tree-based algorithms, which adopt different ways to construct trees or employ different schemes to evaluate feature importance. mRMR evaluates feature importance in terms of its relevance to targets and redundancies to other features. Accordingly, they can investigate a given dataset from different viewpoints. We applied all these algorithms to the investigated dataset. This operation can widely assess feature importance, thereby reducing the probability of missing key features. Brief descriptions of the above algorithms are given as follows.

#### 2.2.1. Least Absolute Shrinkage and Selection Operator

The LASSO [17] algorithm is a regularized linear regression technique that applies an L1 penalty to the regression coefficients. The penalty term is proportional to the absolute values of the coefficients: When the regularization parameter λ is large, many of the coefficients will shrink toward zero, and some may even become exactly zero. Features with non-zero coefficients in the final model are considered important. The magnitude of the coefficients can indicate the relative importance of the retained features. A larger coefficient indicates that the feature is more important and has a greater influence on predicting the target variable.

#### 2.2.2. Monte Carlo Feature Selection

The MCFS [18] algorithm is a robust, versatile method to evaluate the importance of features in datasets of a complex structure—most notably in high-dimensional scenarios where there are more features than observations. MCFS is based on the principles of randomization and ensemble learning. This method uses the advantages of decision tree and random sampling to identify the features that can best predict the target variables. Different from the traditional feature selection method, MCFS constructs multiple decision trees on random subsets of features and data, thus capturing the interaction between features in different contexts. The core idea is that a feature is considered important if it is involved critically in tree constructions.

#### 2.2.3. Minimum Redundancy Maximum Relevance

The mRMR [19] algorithm is a powerful and effective feature selection technique that seeks to identify the most relevant features for a given target variable while avoiding redundancy between the features. The relevance and redundancy are both quantified by mutual information. It is especially useful for high-dimensional data, where feature selection plays a vital role in improving model performance and interpretability. By balancing relevance and redundancy, mRMR is a robust method to rank features, thus improving prediction accuracy and model efficiency.

#### 2.2.4. CatBoost

The CatBoost [20] algorithm, being a Gradient Boosting algorithm, builds an ensemble of decision trees to make predictions. These trees help evaluate the impact of each feature on the target variable, and we can quantify and visualize this importance. The default method used by CatBoost to compute feature importance is prediction change importance. This method computes how much the prediction performance changes when the feature values are randomly permuted (shuffled), which helps identify the contribution of each feature to the model’s predictions.

#### 2.2.5. XGBoost

The XGBoost [21] algorithm is an advanced implementation of Gradient Boosting that is very popular for machine learning tasks, especially in structured/tabular data. Feature importance can be estimated with weight importance in XGBoost. This method counts how often a feature is used in the decision trees to split the data. A feature is rated more important if it has been used more often in the process of tree construction—it is used in more splits. It provides a way to find out which features take part more often in building trees, but it does not consider anything about the quality or effectiveness of the splits, which are created by the feature.

#### 2.2.6. AdaBoost

The AdaBoost [22] algorithm is one of the most popular ensemble learning algorithms to boost the accuracy of weak classifiers. An effective classifier is constructed by combining multiple weak classifiers (in the case of decision trees with a single split, called “stumps”). AdaBoost iteratively adjusts the weight of every training sample according to the current classifier performance and is therefore an adaptive algorithm, emphasizing more on misclassified samples. The key idea is to pay attention to samples that are difficult to classify, improving the model performance over time. As far as the importance of features is concerned, the features that frequently appear in weak classifiers, especially in the early iterations where the model learns the most, will be given higher importance. In contrast, features that are not often used or do not help to reduce classification errors are considered less important.

#### 2.2.7. Random Forest

The RF [23] algorithm is a powerful and versatile ensemble learning technique, which combines a set of decision trees to improve the predictive performance. The underlying idea of random forest is to combine the predictions of a sequence of decision trees, which are trained according to random data subsets and feature subsets. This reduces variance and helps to prevent over-fitting compared with a single decision tree. Every tree in the forest makes an independent prediction, and the final prediction is obtained by aggregating the prediction results of all trees. In random forest, the importance of features is determined by calculating the contribution of each feature in reducing the impurity (or error) of the model. The most important features are those that provide the highest information gain and help most to reduce data uncertainty.

#### 2.2.8. ExtraTrees

The ExtraTrees [24] algorithm is another ensemble algorithm similar to random forest, but the difference lies in decision tree formation. ExtraTrees randomly chooses the split threshold (decision boundary) for each attribute, as opposed to more deterministic methods such as random forest, e.g., Gini impurities or entropy minimization. Additional tree algorithms are designed to improve the randomness and efficiency of the trees in the ensemble, which can lead to faster training and potentially better generalization. Similar to random forests, ExtraTrees can also generate feature importances that indicate how relevant each feature is to prediction. The importance can either be calculated by a decrease in impurity (Gini impurity or entropy) or by permutation importance.

#### 2.2.9. LightGBM

The LightGBM [25] algorithm is one of the most popular and efficient open-source implementations of the Gradient Boosting Machine framework, with objectives to enhance speed, scalability, and support for distributed computing. LightGBM, developed by Microsoft, is optimized for performance that is especially suitable for large datasets and complex machine learning tasks, such as classification, regression, and ranking. LightGBM is a tree-based model, which provides several methods to evaluate the importance of the features according to their contribution in constructing the whole model. The most commonly used method to measure the importance of features is Split Importance. This method measures the total number of times a feature is used to divide nodes in all trees. Features that are often selected for splitting in decision trees are considered more important.

#### 2.2.10. Ridge Regression

The Ridge [26] algorithm is also known as L2 regularization because it adds the L2 norm (the sum of squared coefficients) as a penalty term to the least squares objective function. Thus, it shrinks the coefficients of less important features and produces more stable, generalized models. This technique may be particularly useful when you have a large number of features, many of which may be correlated or not informative. Because ridge regression will not completely eliminate the features (unlike LASSO regression, which can set the coefficients to zero), the importance of the features is usually determined by the magnitude of the coefficients. A larger coefficient indicates that the corresponding feature has a higher importance in predicting the target variables, while a smaller coefficient indicates a lower contribution.

As mentioned above, all the above feature ranking algorithms were applied to the investigated dataset, resulting in some feature lists, which sorted features according to their importance. The obtained feature lists were called the LASSO, MCFS, mRMR, CatBoost, XGBoost, AdaBoost, RF, ExtraTrees, LightGBM, and Ridge feature lists. The programs or packages of the above algorithms were retrieved from public resources, which are listed in Table S1. Default parameters were adopted for all programs or packages.

### 2.3. Incremental Feature Selection

IFS [15] is a feature selection technique that selects the most important features for a given classification algorithm. Based on a feature list, IFS constructs a lot of feature subsets using a step *s* in a way that the first subset contains the top *s* features, the second subset contains the top 2*s* features, and so forth. A classifier is built using the given classification algorithm on each feature subset, which is evaluated by cross-validation [27,28,29,30]. Then, it finds the classifier yielding the best performance, measured by weighted F1 in this study, and the feature subset for this classifier is picked up as the optimal feature subset. The main objective of IFS is to enhance the prediction performance of a classification algorithm by selecting the most informative features and discarding those features that are irrelevant or redundant.

### 2.4. Synthetic Minority Over-Sampling Technique

Synthetic Minority Over-sampling Technique (SMOTE) [31] is a popular technique to solve the problem of class imbalance in machine learning tasks, especially in classification. SMOTE does not simply copy existing instances of minority classes (such as in basic over-sampling) but generates synthetic samples similar to existing minority class samples. It first randomly selects one sample, say *x*, from a minority class. The distances between the selected samples and other samples in the same class are calculated, thereby selecting *K*, a predefined parameter, most similar neighbors. One neighbor is randomly selected, denoted by *y*. A synthetic sample *z* is generated by *x* and *y* in the following manner:

(1) z=a·x+(1−a)·y,

where *a* is a random number between 0 and 1. This synthetic sample is put into the minority class to enlarge its size. The above procedures can execute several times until the dataset is balanced.

In this study, the SMOTE program was obtained from a public resource, listed in Table S1. Default parameters were used to perform this program. Before training the classifiers, the investigated dataset was processed by SMOTE such that each class contained an equal number of samples. In detail, each class finally contained 47 samples, which correspond to the number of samples in the largest class (MenACWY-onset group).

### 2.5. Classification Algorithms

In IFS, a classification algorithm is necessary. Clearly, different classification algorithms may yield different optimal feature subsets. For extracting the optimal feature subset as important as possible, we employed 12 classification algorithms in this study, of which 5 feature ranking algorithms (AdaBoost, RF, ExtraTrees, LightGBM, and Ridge) can also be used as classification algorithms. The remaining seven classification algorithms are briefly described below.

#### 2.5.1. Decision Tree

The DT [16] algorithm is a popular supervised machine learning algorithm. It uses tree structure to model data, in which nodes represent features or attributes in the dataset; edges represent decision rules based on these features; leaf nodes represent output or target variables (for example, class labels or continuous values). The tree construction process starts from the root node containing the whole dataset, and at each internal node, the dataset is split by choosing the best feature (attribute) that divides the data into subsets. The goal is to create homogeneous subsets to achieve more accurate prediction. Splitting continues recursively until one of the stopping criteria is met, for example, when all data points belong to the same class, reaching the maximum depth, or it is impossible to obtain a further significant split. Decision trees are transparent, and their decisions can be visualized. However, the tree may become too complex and capture noise, resulting in low performance for novel data.

#### 2.5.2. K-Nearest Neighbors

The K-nearest neighbors (KNN) [32] algorithm is a simple, intuitive, and widely used supervised machine learning algorithm. For a new data point (query point), KNN calculates the distance between the query point and all points in the training set. The algorithm identifies k nearest neighbors of the query point using distance metrics, such as Euclidean distance, Manhattan distance, and Minkowski distance. For classification, the algorithm assigns the most frequent class label among the k nearest neighbors as the class label of the query point. Although it is very flexible and easy to implement, its computational overhead may be very high for large datasets.

#### 2.5.3. Support Vector Machine

Support vector machine (SVM) [33] is a powerful and versatile supervised machine learning algorithm. The main goal of SVM is to find the best boundary (or optimal hyperplane) to separate data points by class in the feature space. The optimal hyperplane is the one with the largest margin between two classes of data points. Margin is defined as the distance between the hyperplane and the nearest data points of the two classes. These nearest data points are called support vectors, and they determine the optimal boundary. In many real cases, data is not linearly separable. SVM solves this problem by using a technique called the kernel trick. The kernel trick maps the original data points to a higher dimensional space, in which linear hyperplanes can be used to separate data. SVM is especially famous for its ability to process high-dimensional data and its effectiveness in classifying data when the number of features exceeds the number of data points.

#### 2.5.4. Nearest Centroid Classifier

The Nearest Centroid Classifier [34] is a simple and interpretable supervised machine learning algorithm. It assigns a class label to a data point based on the distance between the data point and the centroids (mean vectors) of each class in the feature space. It is often used when computational efficiency and simplicity are important, and it can perform well in scenarios where classes are well-separated.

#### 2.5.5. Stochastic Gradient Descent Classifier

The stochastic gradient descent (SGD) classifier [35] is a linear classifier, which uses stochastic gradient descent as the training optimization technique. The gradient descent is an optimization algorithm to minimize the loss function (or cost function). Through iterative adjustment of model parameters (weights), gradient descent aims to find the best parameters to minimize losses. SGD is a variant. Instead of using the whole dataset to calculate the gradient of the loss function, it updates the parameters of the model after processing each individual training example. This makes the update faster and more efficient, especially for large datasets.

#### 2.5.6. Naïve Bayes Classifier

The Naïve Bayes Classifier [36] is a probabilistic machine learning algorithm based on Bayes’ Theorem. It is used for classification tasks, especially in cases where the input features are independent of each other, a simplifying assumption known as conditional independence. Despite its simplicity, the Naïve Bayes Classifier often performs surprisingly well, particularly in applications like text classification (e.g., spam filtering, sentiment analysis) and other tasks where the features are relatively independent.

#### 2.5.7. Quadratic Discriminant Analysis Classifier

Quadratic Discriminant Analysis (QDA) [37] is a classification technique for machine learning and statistics. It establishes a probability model to classify data into multiple categories. It is a variant of Linear Discriminant Analysis (LDA), and the main difference is that QDA assumes that each class has its own covariance matrix, while LDA assumes that all classes share the same covariance matrix. QDA works on Bayes’ theorem, which, given a data point and its feature values, calculates the posterior probability of that point belonging to each class. The boundaries between categories are quadratic since they depend on quadratic terms of the discriminant function. QDA is a powerful tool in classification, as the model can capture complex decision boundaries; however, it does require accurate covariance estimation, hence limiting its applicability in some areas.

The packages of 12 classification algorithms were also downloaded from public resources, which are presented in Table S1. For convenience, they were executed using default parameters.

### 2.6. Performance Evaluation

In this study, the weighted F1 was used to evaluate the performance of the classifiers [38,39,40,41]. The weighted F1 is commonly employed in the assessment of multi-class classification problems. It is the weighted average of the F1 score (F1-measure) for each class, with the weights corresponding to the frequency of each class in the dataset (i.e., the proportion of samples belonging to each class). When there is a significant disparity in the number of samples across different classes in a multi-class problem, the weighted F1 helps mitigate bias arising from class imbalance and provides a more comprehensive evaluation of the model’s performance. The formula is as follows:

(2) $${Precision}_{i}=\frac{{TP}_{i}}{{TP}_{i}+{FP}_{i}},$$

(3) $${Precision}_{weighted}=\sum_{i=1}^{L}{Precision}_{i}\times w_{i},$$

(4) $${Recall}_{i}=\frac{{TP}_{i}}{{TP}_{i}+{FN}_{i}}\;,$$

(5) $${Recall}_{weighted}=\sum_{i=1}^{L}{Recall}_{i}\times w_{i},$$

(6) $$Weighted\;F1=\frac{2\cdot {Precision}_{weighted}\cdot {Recall}_{weighted}}{{Precision}_{weighted}+{Recall}_{weighted}},$$

In this formula, *i* represents the index of an individual class, *w_i_* denotes the proportion of samples in class *i* relative to the total number of samples, and *L* refers to the total number of classes. TP, FP, and FN represent true positives, false positives, and false negatives, respectively. This comprehensive formula aggregates the F1 score of each class, enabling a thorough and fair evaluation of the model’s performance, particularly in the presence of class imbalance.

In addition, the macro F1, accuracy (ACC), and Matthews correlation coefficient (MCC) were also used to evaluate the performance of the classification models [42,43,44,45,46,47]. Macro F1 is the average of the F1 score of each class. As it does not consider the different contributions of F1 scores on different classes, it is not a perfect measurement when the dataset is imbalanced. ACC is a commonly used metric in machine learning to assess the correctness of the model’s predictions. However, when there is a significant disparity in the number of samples across different classes in the dataset, ACC may not provide a comprehensive evaluation of the model’s performance, which is similar to macro F1. In contrast, MCC is more suitable for addressing issues related to class imbalance, offering a more balanced measure of model performance. To calculate MCC, we construct matrices *X* and *Y*. The former is used to store the actual class label for each sample, while the latter is used to store the predicted class label for each sample. The MCC formula is as follows:

(7) $$MCC=\frac{cov(X,Y)}{\sqrt{cov(X,X)\cdot cov(Y,Y)}},$$

where cov (·,·) stands for the correlation coefficient of input matrices.

### 2.7. Protein–Protein Interaction Network Prediction and GO Enrichment Analysis (Biological Process)

The identified genes were submitted to the STRING online database (http://string-db.org, accessed on 7 January 2025). Based on human homologous protein, the protein–protein interaction (PPI) network of the target gene was predicted and constructed with the medium confidence parameter (0.4). Proteins that did not interact with other proteins were removed. At the same time, the GO enrichment analysis (biological process) of the identified genes was completed by using the STRING online database.

### 2.8. Outline of the Analysis Procedure

**Figure 1:**
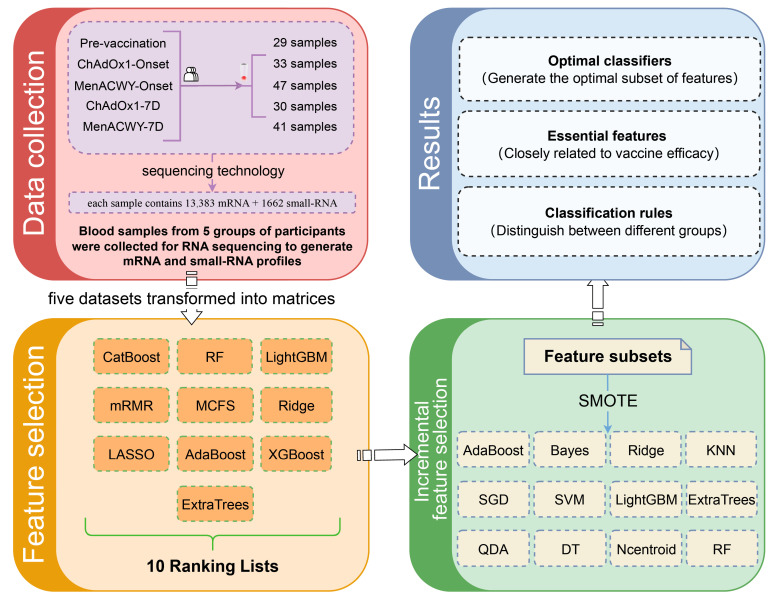
Flowchart of the analysis process. The analysis process was divided into four main parts: data collection, feature selection, incremental feature selection, and results. In the data collection phase, the data was first grouped and pre-processed. The processed data was then input into ten feature ranking algorithms to generate feature ranking lists. This step is referred to as feature selection. In the incremental feature selection phase, the study combined the Synthetic Minority Over-sampling Technique (SMOTE) with twelve classification algorithms to process the feature lists. Using these techniques, three key outcomes were ultimately extracted: essential features, classification rules, and the optimal classifiers.

This study presents a machine learning approach for analyzing gene expression differences between persons who have received the ChAdOx1 nCoV-19 vaccine and those who have not. The research workflow is shown in Figure 1. First, we employed the original data from Drury RE et al. [11] and divided the data into five groups according to different sampling times, vaccination status, and SARS-CoV-2 infection status. The data were derived from RNA sequencing of blood samples from the participants, with each sample containing 13,383 RNA features and 1662 small RNA features. Next, we applied ten feature ranking algorithms to generate ten different feature lists. These feature lists were subsequently fed into the IFS method. By combining the SMOTE and processing with twelve classification algorithms, we ultimately identified key features (including genes and small RNAs) and classification rules associated with the immune response induced by the ChAdOx1 nCoV-19 vaccine. Optimal efficient classifiers were also constructed based on the key features.

## 3. Results

### 3.1. Feature Ranking Results

In this study, the following 10 feature ranking algorithms—AdaBoost, CatBoost, ExtraTrees, LASSO, LightGBM, MCFS, mRMR, RF, Ridge, and XGBoost—were used to deeply analyze the 13,383 RNA and 1662 small RNA features contained in each of the 180 samples. All 10 algorithms ranked features according to different evaluation criteria, resulting in 10 distinct feature lists. The features were ranked based on their importance, with those assigned a higher rank being considered more critical features. The results are detailed in Table S2. To simplify the discussion, the lists obtained from the ten algorithms will be referred to as the AdaBoost, CatBoost, ExtraTrees, LASSO, LightGBM, MCFS, mRMR, RF, Ridge, and XGBoost feature lists. These results provide valuable insights for further investigation and understanding of the immune response induced by the ChAdOx1 nCoV-19 vaccine.

### 3.2. Results of IFS with Different Classification Algorithms

**Figure 2:**
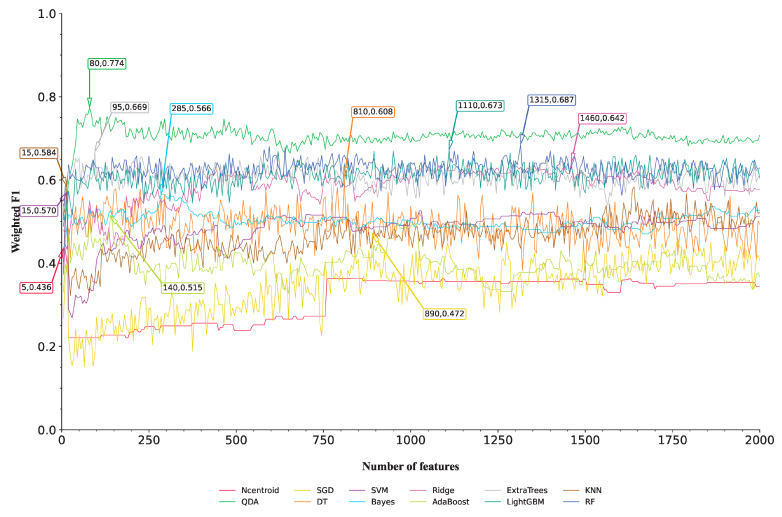
IFS curves for displaying the performance of twelve classification algorithms on the AdaBoost feature list. The QDA achieved the highest performance at the first 80 features, with a weighted F1 of 0.774. The horizontal coordinate represents the number of features used in the feature list, and the vertical coordinate denotes the weighted F1. Different colored curves correspond to different classification algorithms, and the number marked represents the number of features corresponding to that position.

In Section 3.1, ten feature lists were obtained, where features were ranked based on different criteria. The IFS method was applied to each feature list. Since the essential RNA and small RNA features are too many, only the top 2000 features in each list were considered. Furthermore, we adopted step five to construct feature subsets to accelerate the IFS procedure. For each constructed feature subset, twelve classifiers using twelve classification algorithms were built. In total, 4800 classifiers were generated on each feature list, and their performance was evaluated through ten-fold cross-validation. The detailed results can be found in Tables S3–S12. To compare the performance of different classifiers, we plotted IFS curves for each classification algorithm. The vertical axis represents the weighted F1, while the horizontal axis represents the size of the corresponding feature subset (i.e., the number of used features). The IFS curves on the AdaBoost feature list are illustrated in Figure 2, whereas those on the other nine feature lists are shown in Figures S1–S9. By examining the IFS curves, we can visually assess the impact of the number of features on the classifier performance and use this information to select the optimal feature subsets.

By analyzing the IFS curves on the AdaBoost feature list (Figure 2), we found the highest weighted F1 values yielded by each classification algorithm, which are marked on the corresponding curves in Figure 2, along with the number of used features. For example, QDA yielded the highest weighted F1 of 0.774 when the top 80 features were used. Accordingly, the best QDA classifier on the AdaBoost feature list can be constructed using these top 80 features. The best classifiers with the other classifications can be obtained in a similar way. The detailed performance of these best classifiers can be found in Table S13. It can be found that the best QDA classifier was superior to the other best classifiers. Thus, we termed this classifier as the best classifier on the AdaBoost feature list, whereas the features used in this classifier (top 80 features in the AdaBoost feature lists) were regarded as the optimal features identified by AdaBoost.

A similar analysis was conducted on the IFS curves on the other nine feature lists (Figures S1–S9). We obtained the best classifiers using the different classification algorithms on each feature list. Their performance is listed in Table S13. Then, the best classifier on each feature list was found, and the optimal features on each feature list were extracted. The performance of the ten best classifiers on the ten feature lists is listed in Table 2. By comparing these classifiers, it can be found that the best classifier on the LightGBM feature list yielded the highest ACC, MCC, macro F1, and weighted F1. All these measurements exceeded 0.84, implying the high performance of this classifier. This was a useful tool to classify the blood samples into five groups.

### 3.3. Intersection of Essential Features Identified by Different Feature Ranking Algorithms

With the above arguments, the best classifiers on the ten feature lists were constructed. Accordingly, the features used in these classifiers (i.e., the optimal features) were deemed to be important. However, sometimes there were too many optimal features. For example, there were 1685 optimal features extracted from the Ridge feature list. This induced difficulties for further analysis. Thus, we had to extract more important features from them. In view of this, we carefully checked the IFS results on the CatBoost, ExtraTrees, LightGBM, MCFS, mRMR, Ridge, and XGBoost feature lists, trying to find out the classifiers that used much fewer features but provided similar performance to the best classifiers. For convenience, these classifiers were called the suboptimal classifiers. As for the best classifiers on the other three feature lists (the AdaBoost, LASSO, and RF feature lists), they needed few features. It was not necessary to find out the above-mentioned suboptimal classifiers. For unified descriptions, the above three best classifiers were also called the suboptimal classifiers. The performance of the suboptimal classifiers on the ten feature lists is provided in Table 3. Compared to the best classifiers, the suboptimal classifiers really needed much fewer features, whereas the performance was slightly lower. For example, the suboptimal classifiers on the Ridge feature list used 60 features, which was much fewer than the optimal features extracted from this list (1685), and yielded a weighted F1 of 0.711, which was 3% lower than that yielded by the best classifier (0.741). Clearly, the features used in the suboptimal classifiers were more essential than the other optimal features. Investigation into these features was helpful to uncover the immune defense mechanisms and inflammatory responses induced by the ChAdOx1 nCoV-19 vaccine.

**Figure 3:**
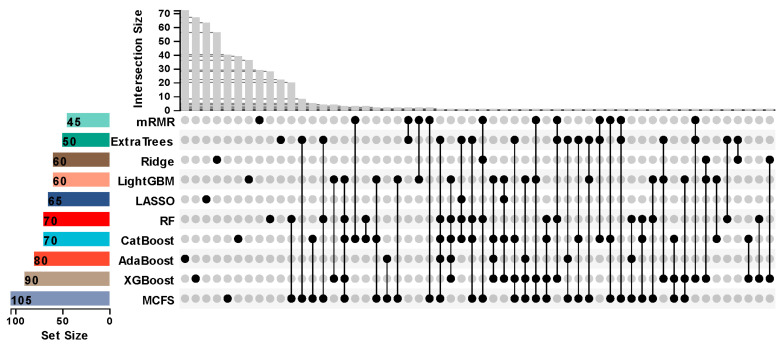
Upset graph of the feature subsets used to construct the suboptimal classifiers on the ten feature lists. “Set Size” is the number of features in each set. “Intersection Size” is the count of the number of features after taking the intersection of some feature sets. The black dots indicate the feature subsets identified by the feature ranking algorithms, and the line between the dots indicates the intersection of some feature subsets.

Ten feature subsets that were used to construct the ten suboptimal classifiers were obtained. By taking the intersection of these feature subsets, an upset graph was plotted, as shown in Figure 3. It can be observed that some features belonged to multiple feature subsets, implying they were identified to be essential by multiple feature ranking algorithms. The detailed features belonging to one, two, or more feature subsets are provided in Table S14. No features belonged to more than five feature subsets, three features (*IGHG1*, *TIMM10*, and *FOXM1*) were in exactly five feature subsets, and ten features (*CASP10*, *AL391244.2*, *HIST1H3G*, *JUN*, *TPX2*, hsa-miR-3614-5p, *EPHB2*, *LGALS3BP*, *ALPL*, *RIOK3*) belonged to exactly four subsets. In this study, we mainly focused on features in multiple feature subsets as they had high probabilities to be essential. As for other features (e.g., features belonging to exactly one subset), they may also be essential, as each feature ranking algorithm has an exclusive ability to discover important features. Thus, we also listed them in Table S14.

**Figure 4:**
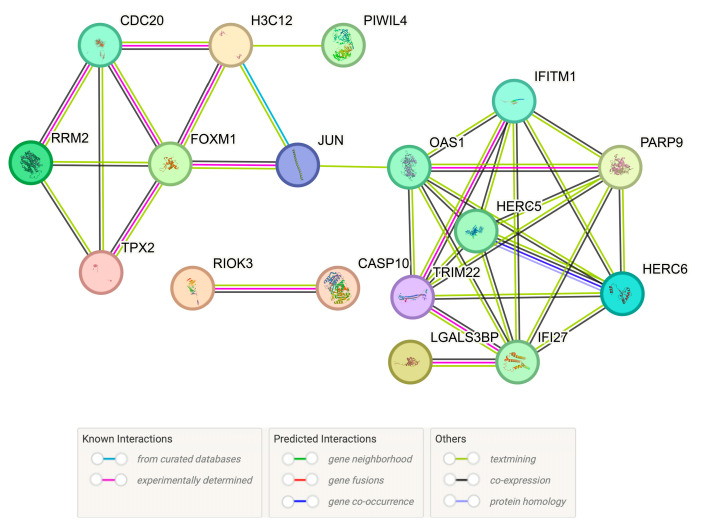
The protein interaction network corresponding to the RNA features in 3/4/5 feature subsets. Proteins that do not interact with any other proteins were removed. The nodes represent different proteins, and the letters labeled next to the nodes are the gene symbols of the corresponding genes. The helical structure inside the nodes is the three-dimensional structure of the protein. The lines between the nodes indicate the interactions between the two proteins, different colors correspond to different types of interactions, there may be one or more interactions between the two proteins, and the detailed types of interactions are shown in the cutline.

We identified several RNA and small RNA features through the above analysis. Further analysis was conducted on RNA features present in three, four, or five feature subsets. First, a PPI network analysis was performed on them to understand the interactions between these gene products. This analysis allowed us to understand whether these gene products work in concert or participate together in specific biological processes and to look for key nodes in the interaction network. These nodes may play important roles in vaccine-induced immune protection, and in-depth analysis with these nodes helped us to enhance our understanding of the function and coordination mechanisms of the immune system during vaccine immune response. The results are shown in Figure 4.

**Figure 5:**
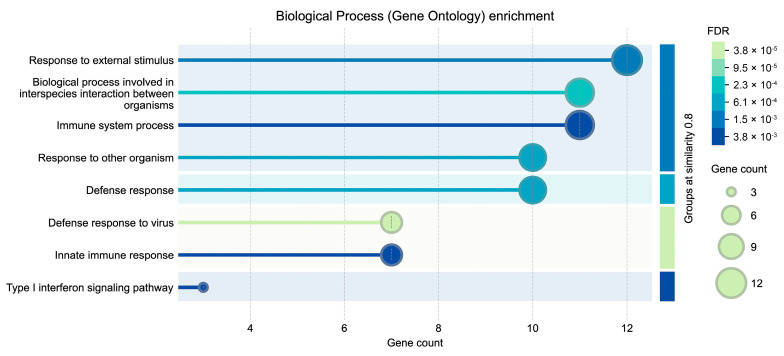
GO enrichment analysis (biological process) of RNA features in 3/4/5 feature subsets. The vertical axis represents the enriched GO terms, and the horizontal axis shows the gene count for each term. The color intensity of the bubbles reflects the FDR value, with lighter colors indicating smaller FDR values (higher significance) and darker colors indicate larger FDR values. Larger bubbles indicate higher gene counts for that GO term, and smaller bubbles indicate lower gene counts for that GO term.

Furthermore, a deeper analysis of the biological functions related to RNA features in three, four, or five feature subsets was conducted, which can give rise to better insights into the basic immune mechanisms involved. GO enrichment analysis (biological processes) was performed on these features to reveal the biological significance behind them (Table S15, Figure 5). GO analysis systematically revealed the biological processes in which these genes are jointly involved with statistical significance. It allowed us to know which biological activities these key genes are mainly involved in and, thus, indirectly infer the immunoprotective mechanism of the ChAdOx1 nCoV-19 vaccine against COVID-19. In addition, GO analysis provided a direct biological mechanistic explanation of the interactions found in the PPI network. The results indicate that the key genes identified by multiple feature ranking algorithms are predominantly involved in biological stress responses and immune processes. The GO terms with the highest gene count included “response to external stimulus,” “biological process involved in interspecies interaction between organisms,” “immune system process,” “response to other organism,” and “defense response”. These GO terms related to immunity, defense, and response to external stimuli further validate the importance of the key genes in the immune response mediated by the ChAdOx1 nCoV-19 vaccine. Among them, the terms “response to external stimulus”, “response to other organism”, and “defense response to virus” fully reflect the relevant processes generated by the organism after ChAdOx1 nCoV-19 vaccination to counteract the invasion of exogenous substances, demonstrating the link between these key genes and vaccine efficacy. Understanding these biological processes helps to indirectly infer the immunization mechanism of ChAdOx1 nCoV-19 vaccine against COVID-19.

### 3.4. Classification Rules Created by Decision Tree

DT was adopted in the IFS method. Although the best DT classifiers on the ten feature lists did not provide high performance, they can provide additional information for further investigations. DT is a type of white-box algorithm. Its classification procedures are completely open. In detail, rules can be extracted from the tree, each of which indicates a path from the root to one leaf. According to Section 3.2, the features used to construct the best DT classifiers were determined. Based on these features and all the blood samples, ten trees were learnt. Each tree yielded a group of rules, which are provided in Table S16. Each rule contained some conditions, consisting of some features and thresholds, and one result (class). It was clear that the conditions in one rule indicated a special expression pattern of the result of the rule. These expression patterns highlight the differential expression levels of various genes and small RNAs between groups, thereby enhancing our understanding of their roles in the ChAdOx1 nCoV-19 vaccine-induced immune response. Some rules will be discussed in Section 4.

## 4. Discussion

By integrating the conclusions from previously published studies, the analysis and discussion of the results obtained in this study, including key features and classification rule genes, will help us further understand the impact of ChAdOx1 nCoV-19 vaccination on the immune response. This will also enable us to analyze the role of these results in the formation of immune memory and the mechanisms of viral prevention.

### 4.1. Essential Genes Associated with ChAdOx1 nCoV-19 Vaccine Effect Identified by Multiple Feature Ranking Algorithms

**Figure 6:**
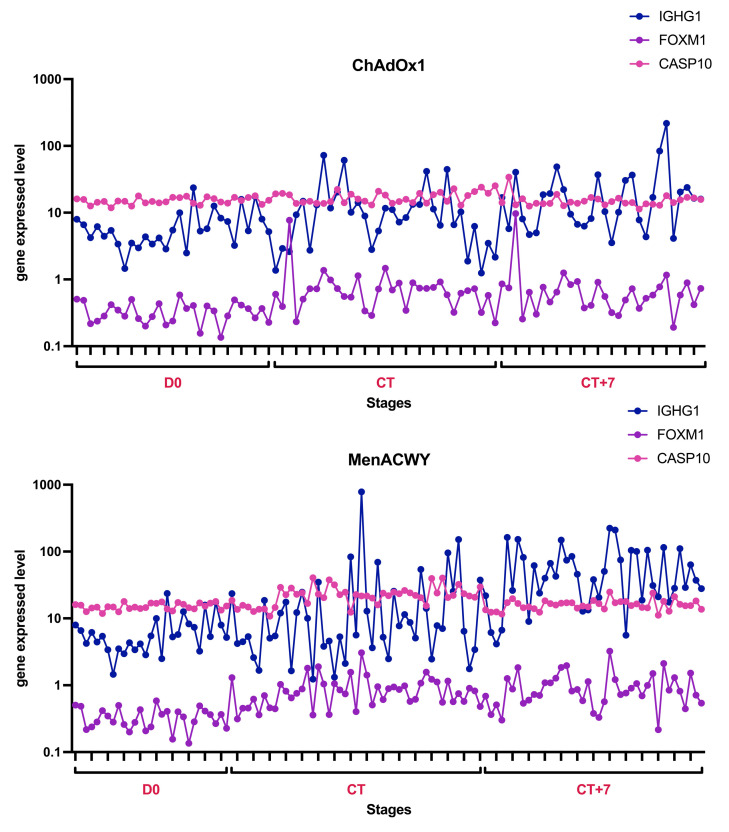
The expression trend of the three genes over time before and after the subjects received different vaccines. The top figure corresponds to the ChAdOx1 nCoV-19 vaccine, and the bottom figure corresponds to the MenACWY vaccine. The *Y*-axis represents the gene expression level, and the *X*-axis represents the stage information. D0: Baseline visit, before vaccination, corresponding to pre-vaccination group. CT: COVID-19 test visit, including ChAdOx1-onset and MenACWY-onset groups. CT + 7: 7 days after CT visit, including ChAdOx1-7D and MenACWY-7D groups. All expression levels were log-transformed by log 10.

**Figure 7:**
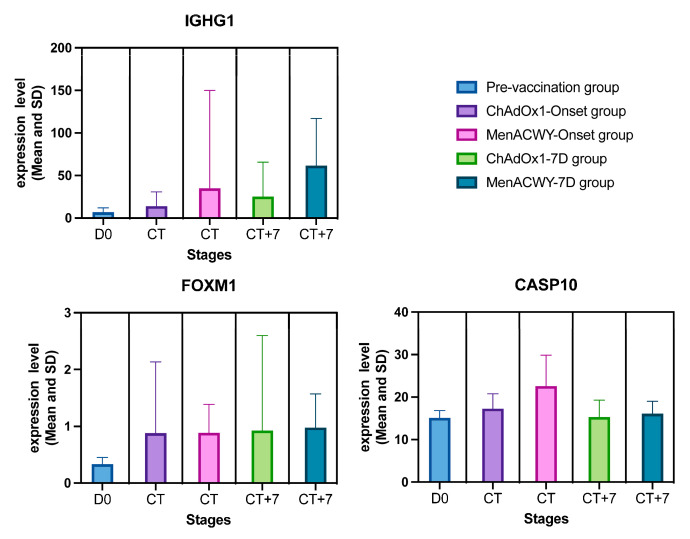
Mean and standard deviation (SD) of *IGHG1*, *FOXM1*, and *CASP10* gene expression in each group. The *Y*-axis represents the gene expression level, and the *X*-axis represents the stage information. D0: Baseline visit, before vaccination, corresponding to pre-vaccination group. CT: COVID-19 test visit, including ChAdOx1-onset and MenACWY-onset groups. CT + 7: 7 days after CT visit, including ChAdOx1-7D and MenACWY-7D groups. The height of each column represents the average gene expression level, and the vertical error bar extension shows the SD of the data.

**Figure 8:**
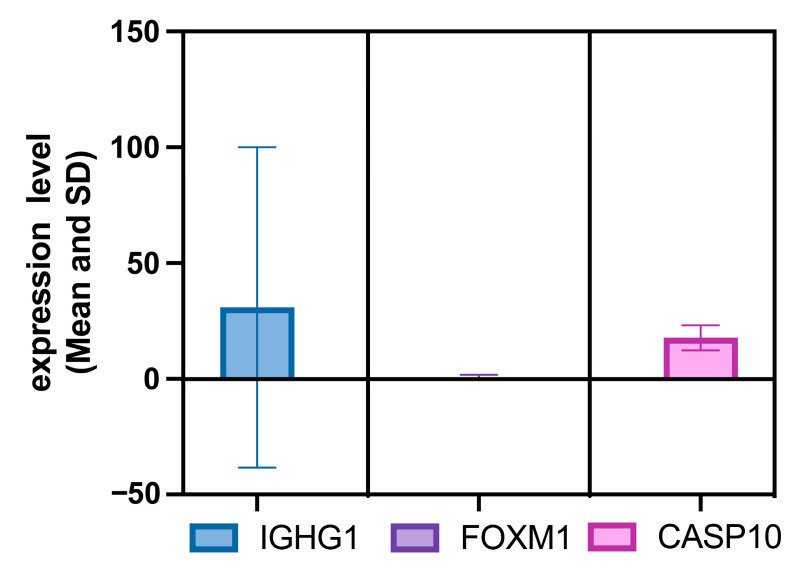
Mean and standard deviation (SD) of IGHG1, FOXM1, and CASP10 gene expression in all samples. The *Y*-axis represents the gene expression level, the *X*-axis represents the different gene information, the height of each column represents the average gene expression level, and the vertical error bar extends up and down to show the SD of the data.

The aim of the study was to use advanced machine learning algorithms to identify key features that indicate the effectiveness of the ChAdOx1 nCoV-19 vaccine. We used AdaBoost, CatBoost, ExtraTrees, LASSO, LightGBM, MCFS, mRMR, RF, Ridge, and XGBoost to sort these features and adopted the IFS method to extract essential features. As shown in Table S14, some genes were identified to be essential by multiple feature ranking algorithms and corresponding classification algorithms. Here, we selected three genes (*IGHG1*, *FOXM1*, and *CASP10*) for analysis, as listed in Table 4. They were identified by at least four feature ranking algorithms. It was shown that these genes are highly correlated with vaccine-induced immune response. We also plotted the expression of the three genes over time (Figure 6) and plotted the mean and standard deviation (SD) of the expression of the three genes in each group of samples as well as in all the samples, respectively (Figure 7 and Figure 8), which were used to show the concentration trend (mean) and dispersion (SD) of the data. Focusing on these key genes will help us to further understand the gene expression changes and immune response induced by ChAdOx1 nCoV-19 vaccine.

#### 4.1.1. Role of *IGHG1*

Immunoglobulin heavy constant gamma 1 (IGHG1) is a subclass member of immunoglobulin G (IgG), a critical component of the human immune system, and an important functional isoform of IgG [48,49,50]. The expression of *IGHG1* encodes the constant region of the heavy chain of IgG. In certain cancers, including pancreatic cancer, glioblastoma, and prostate cancer, *IGHG1* is associated with immune evasion by cancer cells and immune cell infiltration [48,51,52]. A study on glial cancer has shown that *IGHG1* can play an interfering role in the immune system’s fight against cancer cells, thus inducing immune escape of cancer cells, making the expression of *IGHG1* negatively correlated with the survival rate of patients and their prognosis [51,53]. Another study showed that in pancreatic cancer, *IGHG1* can reduce the cytotoxic activity of NK cells by inhibiting antibody-dependent cellular cytotoxicity function and ultimately producing the effect of contributing to cancer cell proliferation and immune escape [48]. In addition, studies on other diseases have also shown that *IGHG1* is involved in the body’s immune regulation process. *IGHG1* expression is increased in antibody-mediated renal allograft rejection [54]. In blood samples of systemic lupus erythematosus, an autoimmune disease, *IGHG1* was highly expressed in B cells [55], suggesting that *IGHG1* can indirectly or directly affect the interaction between cancer cells and the immune system. The results of GO enrichment analysis (Figure 5), with GO terms featuring high gene counts such as “response to external stimulus”, “immune system process”, and “defense response”, further corroborate the findings of IGHG1’s involvement in tumor immune evasion, immune cell infiltration, and antibody-mediated immune functions. These results collectively highlight *IGHG1* as a potential key molecular target in immune response and immune regulation. In the five groups of samples in this study, the expression of *IGHG1* fluctuated (SD) (Figure 6 and Figure 7), and the overall expression level (mean) (Figure 8) was high. Among them, the expression level of *IGHG1* in the pre-vaccination group was lower and more stable compared with the other four groups, suggesting that *IGHG1* may play an important role in the immune response generated by the organism after infection with SARS-CoV-2. In addition, the mean value of *IGHG1* expression was higher and fluctuated more in the MenACWY-onset group and MenACWY-7 D group compared to the ChAdOx1-onset group and ChAdOx1-7D group. This may be due to the lack of ChAdOx1 nCoV-19 vaccine exerting immunoprotection in the MenACWY group, resulting in the body relying on itself to generate a stronger immune response to viral invasion. The mean *IGHG1* expression levels were higher in both the ChAdOx1-7D and MenACWY-7D groups (CT+7 in Figure 7) than in the ChAdOx1-onset and MenACWY-onset groups (CT in Figure 7), respectively, during the infection time period in the ChAdOx1 and MenACWY groups. This suggests that high *IGHG1* levels are associated with a longer recovery period and that they are involved in the body’s long-term immune memory of viral infection, which is consistent with the findings of Cong Lai et al. [74]

#### 4.1.2. Role of *FOXM1*

Forkhead box M1 (FOXM1) is an important member of the forkhead transcription factors family [56], which promotes the transition of G1-S and G2-M cell cycles and is expressed in all proliferating cells [57]. As an important proliferation-related transcription factor, *FOXM1* is highly expressed in most cancers and plays an important role in cell cycle and cell growth [57,58,59,60,61,62]. In addition, *FOXM1* is also involved in the regulation of immune cells [63,64,65]. Most studies have shown that the expression of *FOXM1* is highly correlated with the expression of PD-L1, a transmembrane protein related to immune system suppression [66,67]. FOXM1 can up-regulate the expression of PD-L1 by binding to the PD-L1 promoter in the nucleus [68], and FOXM1-peptide proteolysis targeting can decrease the expression level of PD-L1 protein, suggesting that *FOXM1* can indirectly participate in cellular immune regulation through PD-L1. This further verified the reliability of GO terms such as “organisms Immune system process” and “Innate immune response” in the GO enrichment analysis results. In a study on diabetic foot ulcer (DFU), it was also found that the mediator of macrophage and neutrophil recruitment is FOXM1, which is responsible for the activation and recruitment of inflammatory cells and has the function of activating and promoting the survival of immune cells. Inhibiting FOXM1 inhibits the growth of immune cells and delays wound healing [69]. A study on hepatocellular carcinoma has also shown that *FOXM1* is involved in promoting the process of immune cell infiltration in tumors [70]. It is suggested that *FOXM1* has a potential role in immune regulation. Compared with *IGHG1* and *CASP10*, the expression level of *FOXM1* was very low. Among them, the lowest expression level of *FOXM1* was found in the pre-vaccination group (D0 in Figure 7), and there was no significant difference in the mean expression values of the other four groups. However, through the graphs, we can still find certain patterns. For example, the expression means of both the ChAdOx1-7D and MenACWY-7D groups (CT+7 in Figure 7) were higher than the corresponding ChAdOx1-onset and MenACWY-onset groups (CT in Figure 7), although the differences were small. At the same time, the fluctuations in the ChAdOx1-7D and MenACWY-7D groups (CT+7 in Figure 7) were also greater than those in the ChAdOx1-onset and MenACWY-onset groups (CT in Figure 7). These situations were very similar to the expression trend of *IGHG1*. It was hypothesized that the expression was not high because there was no viral invasion at the pre-vaccination stage. After the invasion of SARS-CoV-2, the body rapidly produced an immune response, which led to the up-regulation of *FOXM1* expression. In this process, FOXM may have the effect of activating and recruiting inflammatory cells, activating and promoting the survival of immune cells, and thus accelerating the fight against the virus. The elevated expression of FOXM may also drive cell proliferation to repair damage. It is worth mentioning that, as shown in Figure 6, it was found that both the ChAdOx1-onset group (CT stage) and the ChAdOx1-7D group (CT+7 stage) vaccinated with the ChAdOx1 nCoV-19 vaccine had a surge and then a decrease in the expression of *FOXM1* at the initial stage. This further validates our above conjecture. It suggests that after SARS-CoV-2 infection, the body needs to up-regulate the expression of *FOXM1* to promote the activation of relevant immune cells to maintain the necessary immune response. However, the detailed mechanism of FOXM1 in the initiation of the immune system still needs to be further confirmed by relevant studies.

#### 4.1.3. Role of *CASP10*

Caspase-10 (CASP10) is a member of the cysteine-aspartate protease (caspase) family, which contains 521 amino acid residues, two death effect domains (DED) and two catalytic active subunits, and its main functions are related to apoptosis and cellular immunity. Studies have shown that in the autoimmune disease primary biliary cholangitis (PBC), dysfunction of caspase-10 may lead to cell death and dysregulation of inflammatory response, and the dysregulation of inflammatory response may participate in the pathogenesis of PBC [71]. In a study of hepatocellular carcinoma in rats, caspase-10 was significantly increased in the positively treated group compared to the positive control group and resulted in a decrease in tumor incidence and tumor size. The results suggest that activated caspase-10 in anticancer therapy may participate in the body’s immune process against tumors by activating apoptosis [75]. In addition, the mutation of the *CASP10* gene is one of the causes of type IIA autoimmune lymphoproliferative syndrome (ALPS). As a rare disease, ALPS is characterized by a disturbance of lymphocyte homeostasis, which leads to immune disorders. In this disease, some patients with ALPS have dominant interference mutations in *CASP10* [72], and the decreased function of caspase-10 is related to the defect of the death receptor (DR) signal of various immune cells in ALPS [73]. It is speculated that the dysfunction of caspase-10 can seriously affect the immune regulatory system of the body, resulting in a series of immune diseases. Immunization-related terms in the GO enrichment analysis, such as “organisms Immune system process” and “Innate immune response”, echo these findings. Relevant studies will help to further understand the specific relationship between *CASP10* expression and the immune system. The expression of this gene was generally high and stable in the five groups of samples. Compared with the *IGHG1* and *FOXM1* genes, the expression level of this gene in the pre-vaccination group (D0 in Figure 7) was not significantly lower than that of the other four groups and was even comparable to the expression of both the ChAdOx1-7D and MenACWY-7D groups (CT+7 in Figure 7). It suggests that *CASP10* may not play an important role in immune memory after vaccination. However, at the CT stage (ChAdOx1-onset and MenACWY-onset groups), the expression level of this gene was higher in the MenACWY-onset and ChAdOx1-onset groups, among which it was significantly higher in the MenACWY-onset group. This may be due to the fact that SARS-CoV-2 induces apoptosis in host cells to promote replication and immune system regulation [76]. The organism needs to regulate apoptosis progression by encoding pro- and anti-apoptotic proteins [77], and *CASP10*, as one of the central participants in the apoptotic process, has elevated expression during this process. In contrast, the ChAdOx1-onset group was less affected by the virus and produced less apoptosis because it was vaccinated with the ChAdOx1 nCoV-19 vaccine, resulting in lower *CASP10* expression in the group than in the MenACWY-onset group. In addition, the body needs to produce a stronger immune response against the virus at the initial stage of neocoronavirus invasion, and CASP10 may also be involved in the immune process by activating apoptosis of cells damaged in the process or by maintaining the response of the body’s immune system in other ways.

Overall, the expression of the three genes varied relatively significantly across all samples, with the *IGHG1* gene expression fluctuating the most and having a larger mean value, *FOXM1* having the least and most stable expression, and *CASP10* having a higher and stable expression level. Moreover, the expression trends of each gene also exhibited both similarities and differences among the sample groups. The *IGHG1*, *FOXM1*, and *CASP10* genes were all more highly expressed in the SARS-CoV2-infected onset group than in the uninfected pre-vaccination group, and except for *FOXM1*, the expression of *IGHG1* and *CASP10* in the control MenACWY group was higher than that in the experimental ChAdOx1 group at the corresponding time point. The high expression of the three genes was demonstrated to be a response characteristic of the organism infected with COVID-19, and they can be used as potential biomarkers for predicting infection with COVID-19. Among them, the rising expression of *IGHG1* directly indicated that SARS-CoV-2 successfully activated humoral immunity, and compared with the MenACWY group, the response of the genes in the ChAdOx1 group was weaker, probably because of the help of the ChAdOx1 nCoV-19 vaccine, meaning that these genes do not need higher expression to maintain an immune response. This reflects the good immunoprophylactic effect of ChAdOx1 nCoV-19 vaccine, but the effect of the sample size difference cannot be excluded. On the contrary, the *IGHG1*, *FOXM1*, and *CASP10* genes tended to be more highly expressed in the control MenACWY group without the involvement of the ChAdOx1 nCoV-19 vaccine, which may be due to a stronger immune response generated by the organism but also due to the fact that these subjects were not assisted by the vaccine, thus developing more severe disease. The *IGHG1* gene has been confirmed to have higher expression in severe cases of COVID-19 [74]. Therefore, we believe that the high expression with high volatility of *IGHG1* and *FOXM1* may be an early warning sign of severe COVID-19 disease. In addition, the synergistic high expression and expression volatility characteristics of the *IGHG1*, *FOXM1*, and *CASP10* genes could be used as a potential biomarker to differentiate the vaccination status of ChAdOx1 nCoV-19 in SARS-CoV-2-infected patients. For example, the pattern of sustained ascending expression of *IGHG1* versus post-peak decay of *CASP10* can be used as a diagnostic marker for the staging of disease processes. In addition, the trend of the expression of these key genes can reflect the speed, strength, and persistence of the response of the corresponding pathways and immune mechanisms. Accordingly, it is possible to compare and analyze the omics data of the ChAdOx1 nCoV-19 vaccine with other vaccines. For example, the gradual increase in *IGHG1* expression over time after vaccination with the ChAdOx1 nCoV-19 vaccine demonstrated that the vaccine effectively activated humoral immunity and was associated with the establishment of long-lasting protection after 7 days. The observed up-regulation of *IGHG1* was similarly observed in a transcriptomic study of the mRNA vaccine BNT162b2, suggesting that this marker may be a universal immune activation signal across vaccine platforms [78]. These findings provide a theoretical basis for the development of a multimodal diagnostic system for early warning and real-time monitoring of vaccine response to ChAdOx1 nCoV-19.

### 4.2. Analysis of Decision Rules to Identify Changes in Gene Expression Resulting from COVID-19 Vaccination

The following section presents four classification rules to differentiate among the pre-vaccination group, the ChAdOx1-onset group, the MenACWY-onset group, the ChAdOx1-7D group, and the MenACWY-7D group. We will provide an in-depth analysis of the key parameters in these four rules, in which no small RNA is included. The validity of these rules is supported by the recent literature, and by examining the key parameters, we provide a comprehensive explanation of the rules.

#### 4.2.1. Rule 0: Distinguishing Between the Pre-Vaccination Group and the ChAdOx1-Onset Group

The first rule is rule 0, which is the rule that distinguishes the pre-vaccination group from the ChAdOx1-onset group and consists of two genes, *HIST1H3G* and *CASP10*. The first gene, *HIST1H3G*, is down-regulated in the pre-vaccination group and up-regulated in the ChAdOx1-onset group. HIST1H3G, also known as H3 clustered histone 8 (H3G8), is a member of the histone H3 family. Recent studies have shown that this gene is involved in the body’s immunoregulation and inflammatory response. In a study of diabetic foot ulcer (DFU) infection, researchers found that members of the core histone gene, including *HIST1H3G*, were highly expressed at the beginning of treatment and down-regulated later in treatment, suggesting that *HIST1H3G* may play an important role in host immunity against disease infection [79]. In another study investigating the role of insulin in promoting wound healing, *HIST1H3G* was found to be up-regulated in differentiated macrophages and involved in signaling pathways in the autoimmune disease systemic lupus erythematosus, suggesting a potential role for *HIST1H3G* in the immune system and possibly in wound healing [80]. In addition, it has been reported that histones can exist outside the nucleus and play a role in promoting inflammation and enhancing the body’s defense ability [81,82,83]. This further justifies its use as a key gene in the rule. Differences between the pre-vaccination group and the ChAdOx1-onset group suggest that both the presence or absence of COVID-19-like symptoms, as well as vaccination with ChAdOx1 nCoV-19, may be important factors contributing to the differing trends in *HIST1H3G* expression between the two groups. It is hypothesized that because the pre-vaccination group is not suffering from COVID-19-like disease and has not been vaccinated, the body does not need to up-regulate *HIST1H3G* to play an immune role. However, when the body is affected by a COVID-19-like disease and is vaccinated, the immune system is activated, and *HIST1H3G* may receive the up-regulated immune signals through immunoregulation, thus playing a role in strengthening the body’s defense capacity and participating in the immune process against diseases and infections. The second gene in rule 0 is *CASP10*, which is down-regulated in both groups of this rule. As mentioned above (Section 4.1), caspase-10 (CASP10) plays an important role in cell apoptosis and cellular immunity, and the dysfunction of CASP10 may lead to a disorder of the body’s immune system, thus causing diseases [71]. In addition, caspase-10 abnormalities have been associated with defects in death-receptor-mediated apoptosis of T cells and dendritic cells. It has been shown that hereditary *CASP10* gene mutation is a condition for lymphocyte defects and dendritic cell apoptosis in the autoimmune lymphoproliferative syndrome ALPS [73]. Dendritic cells (DC) are responsible for initiating the immune response to invading pathogens, and their damage will lead to a decrease in immune ability and a disorder of the immune system [84]. COVID-19 infection can lead to a reduction in the number and dysfunction of dendritic cells in the human body. Moreover, the immune escape mechanism of viral proteins encoded by SARS-CoV-2 can further cause confusion in the host immune system [85]. Combined with the above conclusions, we suggest that COVID-19 virus may interfere with the body’s immune system by attacking dendritic cells and other behaviors, resulting in a disorder of the body’s immune system. Disorder of the immune system and the abnormality of dendritic cells are related to the dysfunction of CASP10. It is speculated that the down-regulation of *CASP10* expression in the absence of disease is normal, while the down-regulation of *CASP10* expression in the disease may be one of the processes or results of the immune system disorder caused by COVID-19 infection. However, direct evidence for a specific link between COVID-19 and *CASP10* is still lacking. Relevant studies will be helpful to further verify this conclusion.

#### 4.2.2. Rule 1: Identifying the MenACWY-Onset Group

The second rule, rule 1, for identifying the MenACWY-onset group, has a gene called *ALPL*, whose expression is down-regulated. Alkaline phosphatase (ALPL) encodes a member of the family of ALPL proteins, the tissue-non-specific alkaline phosphatase (TNSALP). Although existing studies have shown that the primary function of this enzyme is related to human bone and tooth mineralization [86], there are still some studies showing the potential role of *ALPL* in immune system regulation. On the one hand, TNSALP regulates purinergic signaling, which can hydrolyze ATP and ADP to produce AMP and adenine and then use adenine receptors to prevent inflammation [87]. On the other hand, TNSALP can also exert its effects through immune cells. TNSALP has been shown to be expressed in phagocytes [88], neutrophils [89], and T lymphocytes. In the tumor microenvironment, CD10^+^ALPL^+^ neutrophils can exhibit strong immunosuppressive activity and induce obvious “irreversible” depletion of T cells [90]. In a study in mice, TNSALP was also involved in the process of stimulation of T lymphocytes and T-cell-dependent colitis [91]. A strong relationship between *ALPL* and COVID-19 has been revealed in other studies. A study demonstrated that *ALPL* was associated with disease severity and pulmonary fibrosis (a major complication of COVID-19) in COVID-19, and by neural network modeling analysis, *ALPL* may be a potential biomarker for identifying the severity of COVID-19 [92]. Another study has measured RNA markers in patients with varying degrees of disease and found that ALPL RNA concentrations were significantly higher in severe cases of COVID-19 than in episodic/moderate cases [93]. In addition, novel COVID-19 drugs are being developed that target *ALPL* as a therapeutic target for the disease [94]. Combined with the above findings, it is hypothesized that the down-regulation of *ALPL* expression in the MenACWY-onset group is related to the MenACWY vaccine. The MenACWY vaccine has a weak activation of intrinsic immunity, a limited effect on the SARS-CoV-2-specific immune response, and just the opposite, although not selected as a key parameter in rule 0, *ALPL* gene expression was up-regulated in the ChAdOx1-onset group. It is speculated that this is due to the fact that vaccination causes the body to produce self-protective mechanisms that mimic the fight against severe neocoronitis. The ChAdOx1 nCoV-19 vaccine activates stronger innate and cellular immunity, which involves a broader range of immune cell types and signaling pathways. Among these, *ALPL* is up-regulated to express TNSALP and prevent inflammation by regulating purinergic signaling. This conjecture is also consistent with the conclusion that *ALPL* serves as a biomarker for severe COVID-19.

#### 4.2.3. Rule 2: Identifying the ChAdOx1-7D Group

The third rule, rule 2, identifies the ChAdOx1-7D group and contains a gene called *IGLL5*, which is down-regulated in the rule. Immunoglobulin-Lambda-Like Polypeptide 5 (IGLL5) is located within the immunoglobulin λ site, and its gene is responsible for encoding one of the IGLLs; although there are few studies on *IGLL5*, the existing research conclusions are still gradually revealing its role in the immune response. In a study of clear cell renal cell carcinoma (ccRCC), it was found that the genes in tumor samples with high *IGLL5* expression were mainly concentrated in immune-related pathways, such as natural killing cell-mediated cytotoxicity, autoimmune diseases, cell adhesion molecules, and chemokine signaling pathways. Moreover, the expression of *IGLL5* was correlated with three types of tumor-infiltrating immune cells (TICs): naive B cells, plasma cells, and activated CD4 memory T cells [95]. Another study showed that *IGLL5* is one of the frequently mutated genes in mature B cell lymphomas [96,97,98]. In this disease, reduced expression of *IGLL5* contributes to apoptosis and reduced expression of MYC [99]. In addition, IGLL5 mRNA was also found to be expressed in mature B cells exhibiting Igκ protein [100]. These results suggest that *IGLL5* may be closely related to immune cells. In this rule, the expression of *IGLL5* is down-regulated, which may be due to the large number of infected cells that need to be eliminated in the process of fighting the virus and the need for *IGLL5* to reduce expression to promote cell apoptosis. On the other hand, it may be that the effect of the vaccine regulates the expression of genes involved in the immune response, so that *IGLL5* is down-regulated in the process. However, further research is needed to confirm this hypothesis.

#### 4.2.4. Rule 3: Identifying the MenACWY-7D Group

The fourth rule, rule 3, identifies the MenACWY-7D group, which has two genes. The first gene is *IGHG1*, whose expression is up-regulated. The gene of immunoglobulin G1 (IGHG1), also known as IgG1, encodes the heavy chain of immunoglobulin G (IgG), and its regulatory effects on the immune system have been described in Section 4.1. At present, studies have explored the impact of COVID-19 infection on *IGHG1* expression, and the research results are consistent with our conclusions, showing that infection with COVID-19 can up-regulate *IGHG1* expression. Studies have found that IgG levels increase significantly and persist for at least eight weeks after the onset of COVID-19 or vaccination [101]. However, in patients with COVID-19 during acute infection, it was mainly IgG1 and IgG3 specific to RBD that had a strong reaction, and IgG2 and IgG4 were difficult to detect in patients’ serum [102,103,104,105]. It is speculated that the immune response to the invasion of SARS-CoV-2 is one of the reasons for the increase in IgG1 levels. In addition, since *IGHG1* is up-regulated 7 days after infection with SARS-CoV-2, it is shown that *IGHG1* produced after infection with SARS-CoV-2 plays an important role in long-term immune memory and helps the body to respond quickly when faced with the same pathogen in the future. The second gene, *IGLL5*, is also included in rule 2, with the difference that its expression is down-regulated in patients vaccinated against ChAdOx1 nCoV-19, whereas it is up-regulated in rule 3. Previous studies have shown that *IGLL5* has a close relationship with immune cells [95,100]. It is speculated that in the case of infection with SARS-CoV-2 without injection of the ChAdOx1 nCoV-19 vaccine, the body needs to produce a strong immune response to resist the virus so that the immune cells in the body, such as B cells and T cells, are activated, and thus the expression of *IGLL5* related to immune cells is up-regulated. On the other hand, the high expression of *IGLL5* 7 days after infection with COVID-19 may be the result of an adaptive immune response, suggesting that this characteristic gene has a potential role in antibody production and immune memory cell formation. The unique expression pattern of *IGLL5* helped us distinguish between the ChAdOx1-7D group and the MenACWY-7D control group.

## 5. Conclusions

In this study, we analyzed RNA and small RNA features using multiple machine learning algorithms to identify essential features induced by ChAdOx1 nCoV-19 vaccination. Subsequently, an efficient classifier with a weighted F1 of 0.871 was constructed, which could effectively discriminate between vaccinated and unvaccinated ChAdOx1 nCoV-19 subjects. Analysis of the essential features revealed that the specific expression patterns of *IGHG1*, *FOXM1*, and *CASP10* could be potential markers characterizing the vaccination status of the ChAdOx1 nCoV-19 vaccine or signals of immune activation for the virus to enter different stages. Finally, the study also constructed classification rules, which can classify samples into five groups of individuals with different ChAdOx1 nCoV-19 vaccination status and indicate special patterns for different ChAdOx1 nCoV-19 vaccination status. These rules promoted the construction of an automated outbreak management system, which helps to quickly triage the population and strengthen outbreak prevention and control. Detailed analysis of the key characteristics and parameters of the rules helps us to understand the immunoprotective effect of the ChAdOx1 nCoV-19 vaccine at the time of symptom onset as well as the immune memory before and after vaccination. This study highlights the potential of machine learning in decoding the molecular mechanisms of the COVID-19 vaccine and provides a theoretical basis for precise prevention and control strategies.

## Figures and Tables

**Table 1:** Sample and feature counts for the five data groups.

Sub-Dataset	Sample Size	Features
Pre-vaccination group	29	13,383 RNA-seq and 1662 small RNA-Seq
ChAdOx1-onset group	33	13,383 RNA-seq and 1662 small RNA-Seq
MenACWY-onset group	47	13,383 RNA-seq and 1662 small RNA-Seq
ChAdOx1-7D group	30	13,383 RNA-seq and 1662 small RNA-Seq
MenACWY-7D group	41	13,383 RNA-seq and 1662 small RNA-Seq

**Table 2:** Performance of the best classifiers on the different feature lists.

Feature List	Classification Algorithm	Number of Features	ACC	MCC	Macro F1	Weigthed F1
AdaBoost feature list	QDA	80	0.753	0.716	0.774	0.774
CatBoost feature list	LightGBM	215	0.750	0.688	0.746	0.750
ExtraTrees feature list	QDA	580	0.736	0.708	0.748	0.748
LASSO feature list	QDA	65	0.719	0.674	0.735	0.735
LightGBM feature list	LightGBM	270	0.872	0.841	0.870	0.871
MCFS feature list	LightGBM	865	0.767	0.709	0.758	0.766
mRMR feature list	QDA	130	0.740	0.704	0.754	0.754
RF feature list	RF	70	0.750	0.690	0.748	0.750
Ridge feature list	QDA	1685	0.723	0.694	0.741	0.741
XGBoost feature list	LightGBM	440	0.844	0.805	0.843	0.843

**Table 3:** Performance of the suboptimal classifiers on different feature lists.

Feature List	Classification Algorithm	Number of Features	ACC	MCC	Macro F1	Weigthed F1
AdaBoost feature list ^$^	QDA	80	0.753	0.716	0.774	0.774
CatBoost feature list	LightGBM	70	0.739	0.674	0.733	0.734
ExtraTrees feature list	QDA	50	0.698	0.675	0.714	0.714
LASSO feature list ^$^	QDA	65	0.719	0.674	0.735	0.735
LightGBM feature list	LightGBM	60	0.833	0.791	0.830	0.832
MCFS feature list	LightGBM	105	0.672	0.593	0.667	0.674
mRMR feature list	QDA	45	0.681	0.657	0.691	0.691
RF feature list ^$^	RF	70	0.750	0.690	0.748	0.750
Ridge feature list	QDA	60	0.698	0.661	0.711	0.711
XGBoost feature list	LightGBM	90	0.811	0.764	0.809	0.809

^$^: These classifiers were the same as the best classifiers on the corresponding feature lists.

**Table 4:** Details of three essential genes found in this study.

Ensembl ID (Gene Symbol)	ENSG00000211896 (IGHG1)	ENSG00000111206 (FOXM1)	ENSG00000003400 (CASP10)
Description	Immunoglobulin heavy constant gamma 1 (G1m marker)	Forkhead box M1	Caspase 10
References	[48,49,50,51,52,53,54,55]	[56,57,58,59,60,61,62,63,64,65,66,67,68,69,70]	[71,72,73]
Corresponding algorithm	CatBoost, RF, XGBoost, MCFS, LightGBM	CatBoost, RF, XGBoost, MCFS, LightGBM	AsaBoost, XGBoost, MCFS, LightGBM
Immunological activity	IGHG1 is a subclass member of immunoglobulin G, which influences the interaction between the immune system and cancer cells, as well as the regulation of immune mechanisms, in certain cancers and immune-related diseases through modulating cancer cell immune evasion, suppressing immune cell function, and others.	FOXM1, as an important member of the forkhead transcription factors family, plays a crucial role in cell cycle, cell proliferation, and immune cell regulation. It can participate in immune regulation by up-regulating the expression of PD-L1 and is closely related to the function of immune cells.	Caspase-10 (CASP10), a member of the cysteine-aspartate protease family, is involved in apoptosis and cellular immunity, with dysfunction or mutations in CASP10 contributing to autoimmune diseases such as primary biliary cholangitis (PBC) and type IIA autoimmune lymphoproliferative syndrome (ALPS), suggesting its critical role in immune regulation.
Pre-vaccination group (mean ± SD)	6.79 ± 4.97	0.33 ± 0.12	15.12 ± 1.70
ChAdOx1-onset→7D group (mean ± SD)	13.77 ± 16.76→25.31 ± 40.09	0.88 ± 1.26→0.92 ± 1.68	17.27 ± 3.49→15.32 ± 3.96
MenACWY-onset→7D group (mean ± SD)	34.77 ± 115.17→61.43 ± 55.69	0.88 ± 0.50→0.98 ± 0.59	22.63 ± 7.24→16.09 ± 2.93
General tendency	Expression and volatility continued to rise over time.	The pre-vaccination group was significantly lower, and the ChAdOx1-/MenACWY-onset and ChAdOx1/-MenACWY-7D groups were similar.	The ChAdOx1-/MenACWY-onset group in-creased over time, while the ChAdOx1-/MenACWY-7D group remained basically unchanged.

## Data Availability

The transcriptomic dataset analyzed in this study was retrieved from Drury et al.’s study [11].

## Supplementary Materials

The following supporting information can be downloaded at: https://www.mdpi.com/article/10.3390/life15060981/s1, Table S1: Details of machine learning algorithms used in this study. Table S2: Feature ranking results generated by AdaBoost, CatBoost, ExtraTrees, LASSO, LightGBM, MCFS, mRMR, RF, Ridge, and XGBoost methods; Table S3: Performance of IFS with different classification algorithms on AdaBoost feature list; Table S4: Performance of IFS with different classification algorithms on CatBoost feature list; Table S5: Performance of IFS with different classification algorithms on ExtraTrees feature list; Table S6: Performance of IFS with different classification algorithms on LASSO feature list; Table S7: Performance of IFS with different classification algorithms on LightGBM feature list; Table S8: Performance of IFS with different classification algorithms on MCFS feature list; Table S9: Performance of IFS with different classification algorithms on mRMR feature list; Table S10: Performance of IFS with different classification algorithms on RF feature list; Table S11: Performance of IFS with different classification algorithms on Ridge feature list; Table S12: Performance of IFS with different classification algorithms on XGBoost feature list; Table S13: Performance of the best classifiers using different classification algorithms on the ten feature lists. Table S14: Intersection of essential gene subsets identified by AdaBoost, CatBoost, ExtraTrees, LASSO, LightGBM, MCFS, mRMR, RF, Ridge, and XGBoost methods on features datasets. The features that appear in the 5, 4, 3, 2, and 1 subsets are shown; Table S15: Information about GO enrichment analysis (biological processes) of genes present in 3, 4, or 5 characterized subsets; Table S16: Classification rules generated by decision tree with its optimal features on the ten feature lists. Each rule is composed of different features and their expression levels and describes how the expression level determines the class labels of samples; Figure S1: IFS curves for displaying the performance of twelve classification algorithms on CatBoost feature list; Figure S2: IFS curves for displaying the performance of twelve classification algorithms on ExtraTrees feature list; Figure S3: IFS curves for displaying the performance of the twelve classification algorithms on the LASSO feature list; Figure S4: IFS curves for displaying the performance of the twelve classification algorithms on the MCFS feature list; Figure S5: IFS curves for displaying the performance of the twelve classification algorithms on the mRMR feature list; Figure S6: IFS curves for displaying the performance of the twelve classification algorithms on the RF feature list; Figure S7: IFS curves for displaying the performance of the twelve classification algorithms on the LightGBM feature list; Figure S8: IFS curves for displaying the performance of the twelve classification algorithms on the Ridge feature list; Figure S9: IFS curves for displaying the performance of the twelve classification algorithms on the XGBoost feature list.

## Abbreviations

The following abbreviations are used in this manuscript:

COVID-19	Coronavirus Disease 2019
SARS-CoV-2	Severe Acute Respiratory Syndrome Coronavirus 2
IFS	Incremental Feature Selection
DT	Decision Tree
LASSO	Least Absolute Shrinkage and Selection Operator
MCFS	Monte Carlo Feature Selection
mRMR	Minimum Redundancy Maximum Relevance
CatBoost	Categorical Boosting
XGBoost	Extreme Gradient Boosting
AdaBoost	Adaptive Boosting
RF	Random Forest
ExtraTrees	Extreme Randomized Tree
LightGBM	Light Gradient Boosting Machine
SMOTE	Synthetic Minority Over-sampling Technique
KNN	K-Nearest Neighbors
SVM	Support Vector Machine
SGD	Stochastic Gradient Descent
QDA	Quadratic Discriminant Analysis
LDA	Linear Discriminant Analysis
ACC	Accuracy
MCC	Matthews Correlation Coefficient
PPI	Protein–Protein Interaction
SD	Standard Deviation
